# Physiology of Spiritual Table Tipping

**Published:** 1853-10

**Authors:** E. Andrews


### SELECTIONS.

From the Peninsular Journal of Medicine.

### *Physiology of Spiritual Table Tipping.* By E. Andrews, M.D.

This veriest humbug that ever exhaled from the caverus of delusion, has brought to light one remarkable physiological truth, viz.: *the power of other functions of the mind besides the will over the muscles.* Physicians should lose no time in investigating it, for another opportunity equally good may not occur in a century. Besides, physicians owe it to society to expose delusions which are based on physiological phenomena, since they alone are competent to do it. They should examine it, therefore, that their exposure may not be the laugh of ignorance, but the piercing sarcasm of men who understand the nature of what they speak.

The Rev. Charles Beecher, of N. J., at the request of an ecclesiastical association, has written a small work on this subject, in which he takes the ground that the " manifestations " are actually the work of evil spirits. His admission of the spirituality of the performances has had a bad effect in this region. All the tipsy tables have tipped with unwonted confidence ever since, and thousands, who held it to be mere nonsense before, are now staggered to learn that a Beecher has decided for the spirits.

We shall give this work a brief review for two reasons : one is, because many physicians who may not see the work itself will, nevertheless, have to combat the influence of its name, and the other is, because it is a fine sample of the pranks cut up by the nervous system, which, no longer content to delude hysterical girls, and superstitious old men and women, has in these last days, bestrode the pulpit, and made a learned divine, one from a family of ecclesiastical giants, think that there is actually a telegraph from the infernal regions, and that we are in the daily receipt of despatches from the devil.

The treatise in question commences, very necessarily, with a statement of the facts. Now, good reader, what kind of a statement of facts do you suppose was made by this clergyman of mighty name, this Beecher, who was appointed by the New York and Brooklyn Association to report on Spiritual Rappings. You who are accustomed to the searching, fact-sifting of scientific bodies—you possibly imagine that he commences with a careful description of the observations and experiments whereby he determined the phenomena, and with a clear statement of the tests whereby he sifted out the error, and analyzed the whole to the ultimate fact elements. Most learned doctor, you are mistaken. The whole question of facts is contained in a dozen lines. Here it is :

"The facts which constitute the pneumatic argument arrange themselves in four classes:—

"Class 1. Mysterious intelligent sounds and movements.

"Class 2. Involuntary polyglott speaking and writing.

"Class 3. Apparitions.

"Class 4. Doctrines, revelations, poems, prophecies and medical prescriptions, all delivered through the above instrumentalities."

This is all he has to say about the facts. The subsequent pages are learned and eloquent, but to what purpose? In science we are accustomed to require our authors to state item by item, all the circumstances in which their facts are observed, and all the tests to which they were subjected, because experience has shown that assertions not thus scrutinized are not worth a fig, and all arguments based on them, though they may be very logical, prove no more than a puff of nonsense.

He next proceeds to do battle with two opposing theories, which he handsomely demolishes, for he is good at the sword exercise of argument. He also brings up "*od*" or "*odyle*" with evident approbation, as the means by which evil spirits effect their communications with men, women and tables, upsetting the latter, jerking the elbows of the former, and kicking up a row generally.

This odyle is a name given to a supposed agent or force, by Baron von Reichenbach, of Vienna. It is supposed to be diffused throughout the universe, and the Baron has written a book of some 400 pages, filled with his observations and experiments upon it. At some future time we may review it for the amusement of our readers, at present suffice it to say that the majority of his phenomena were evidently nothing but the disordered sensations of the "sensitive" and "nervous" subjects upon whom he experimented. The Baron was evidently entirely ignorant of the pathology of the nerves of sensation. Whoever reads his work without understanding physiology will wonder and admire, but a physiologist will wonder and laugh.

The rest of Beecher's essay is devoted to showing that the tipping and rapping spirits are evil and not good spirits.

We have had some acquaintance with ecclesiastical bodies, and we think we can account for the production of this curious document without any disparagement to that noble profession. Clergymen, as a body, are not engaged in discovery. Their business is not so much to investigate new truths, as to enforce old ones; hence, in their associations they assign topics to each other, not so much for the investigation of facts as to develop originality of thought and fire of expression. In all probability the Congregational Association of New York and Brooklyn cared not one fig for the *investigation* of this humbug, but knowing that Mr. Beecher held some peculiar notions upon it, wished to enliven their meeting by drawing forth his ideas and his eloquence.

But laying Beecher aside, what and how much is there in these "manifestations." We have taken some pains to investigate the matter, and we affirm, as the result of our observations, that if you reject one-half of all the accounts for falsehood, and then two-thirds of the remainder for exaggeration and mistake, there will remain the following solid facts:

1st. Disordered innervation, causing delusions of the senses and spasmodic twitching of the muscles in all parts of the body.

2nd. Tables, stands, &c., by placing the medium's hands flat upon the upper surface, may be made to move about the room, get upon a sofa, dance to music, and tip out intelligent answers to questions *without any consciousness of voluntary exertion by the medium.*

3rd. The medium may write communications and answers to questions *without any voluntary directing of the hand or pencil.*

Any one who will seat half a dozen ladies of nervous temperament at a table, and cause them to hold their hands immoveably upon it for an hour, may produce the manifestations of twitching and disordered sensation, and a few repetitions will generally suffice to develop the table-moving power.

The character of many of the mediums is such as to leave no doubt their agency is involuntary, at the same time our investigations have demonstrated the action of the muscles. They usually suppose that with the hands laid flat upon the smooth top of a table, it is impossible for muscular action to take effect, and we have often seen them raise the hand so as only to touch the table with the ends of their fingers and thumbs—a manœuvre which satisfies most observers that there is no muscular action in the case.

Our first experiment was to see whether this position of the hands was a sufficiently rigid test. To our surprise we found that with merely the tips of our fingers touching the table we could imitate all the evolutions of the spirits. We caused it to traverse the room in every direction, made it dance to music, and mount upon the sofa, and with the tip of one finger pressed upon the top of it, we could use force enough to cause it to lie slowly down upon its side, and to rise up again. We next took a circular cherry table, four feet in diameter, and placing our hands flat upon the top, we caused it to walk up the perpendicular walls of our office, and then, top downward, to walk all about the ceiling. Being thus satisfied that muscular power was competent to the effect, we proceeded to test for its actual existence.

The mediums were confident that the tables heaved and moved spontaneously beneath their hand. We placed a sheet of paper beneath their hands, when lo! the table stood still while the paper slid all over it. We next placed a book under their hands, and two round wooden pencils under the book as rollers. The book rolled about with great activity, but the indignant table would not budge an inch. We then placed our own hands under those of the medium, when we could distinctly feel the latter pushing and pulling upon our own. It was evident, therefore, that the power was exerted by the medium, not by the table. Lastly, we placed our fingers upon the tendon of the latissimus dorsi muscle where it crosses the axilla, and having ascertained that it was relaxed, relaxed, requested the spirits to tip, when we could feel the tightening of the tendon as it drew the arm back. The muscular action was decided.

We next proceeded to test by questions, both vocal and mental, by which we ascertained the following facts:

1. When the question was vocal and the medium knew what the answer should be, *the spirit invariably replied correctly.*

2. When the question was such that the medium neither knew the answer, nor could have any possible chance of hitting right by coincidence, *the response was invariably wrong.*

3. When there was a chance of hitting right by coincidence, and in questions of yes and no, and questions of numbers and some others, the answers were sometimes right and sometimes wrong.

4. If the questions were mental, and no chance of guessing right existed, the answers were *always false.* If, in addition, the countenance was so guarded as not to show *when* a mental question was asked, the answers were not only false in substance, but out of time with the question; and answers repeatedly came *when no questions had been asked.*

The following are our notes of one of these dialogues. It was a writing medium of the most unquestionable integrity. Having ascertained that the spirit could answer questions otherwise than by yes and no, we proceeded both with vocal and mental questions.

*Question.* (vocal) Will the spirits communicate?

*Spirit.* Yes.

*Q.* (vocal) Whose spirit it writing now?

*S.* William Bassett's.

*Q.* (mental) William, how many brothers has J——?

*S.* Yes.

*Q.* (mental) Where does the oldest one live?

*S.* No.

*Q.* (mental) Are they both married?

*S.* Yes. (This was incorrect.)
*Q.* (mental) How many sisters has he?
*S.* Yes.
*Q.* (mental) Who married his sister?
*S.* Yes.
*Q.* (vocal) Will the table tip?
*S.* Yes.
*Q.* (vocal) In how many minutes?
*S.* 10. (The spirit used figures.)
*Q.* (vocal) Will the spirit write it in letters?
*S.* Ten. (Here the company went to the table and sat half an hour, but it would not tip.

*Q.* (vocal) What has been performing here to-night? (Here the pencil wrote something which was probable meant for "spirits," but it was nearly illegible and looked more like the word "infernal."

*Medium to the Spirit.* That isn't good; can't you write a little plainer?

The pencil then wrote after the former word very distinctly, S-p-i-r-t-s, "Spirts;" whereupon we took the slate and read the communication to the company, "Infernal Spirts." It was obvious to those present that the "infernal spirts" did not stand very rigid testing.

Putting together these with other facts, therefore it was clear that the knowledge of the medium and the chances of conjecture, had some connection with the correctness or incorrectness of the answers; in short, that the communication came from living souls in this world, not from "infernal spirts" of the other. And yet, the high and irreprochable character of the mediums compelled us to believe that their actions were not voluntary. The question then remained, Can the mental states express themselves in muscular actions *without* the intervention of the will?

The table-tippers and spirit-writers have developed one great truth, which, though previously known in special applications, has only recently seemed to receive a full general acknowledgment, and that is this: Not only the will, but every other function of the mind is a natural stimulant to the muscles, competent, when acting with the will, to give it double effect, but also able to act without it and produce *intelligent involuntary actions.*

Undoubtedly the clearest development of this principle is seen in the muscles of the countenance. These are handed over almost entirely to the involuntary class, and almost all their action is in response to the stimulus of thought and emotion; there is no volition, no consciousness of their action. It is certain, therefore, that thought and emotion, as well as volition, have control over muscular power.

We hold that every muscle in the body is subject to the same influence, and that the reason why we do not notice it, is because the superior powers of volition masks the effects of the other mental functions. If this is true, then we should expect that by giving these functions a relative preponderance ower the will, they would re-assert their motor power and bring the muscles under their control. This may be done by giving the emotions unusual power, as in terror or in pain, the involuntary writhing and recoiling of which are too familiar; or it may be done by concentrating the thoughts on a particular action and withholding the will. This is the method of the mediums, and by it they secure action which corresponds to thought without volition.

Normally, however, this power acts in conjunction with the will. This is the triple strength which nerves the limbs of men under intense excitement—the superadded force which renders them competent to meet great emergencies. We often see at a fire instances where men, with a very slight *voluntary* effort, will pick up and carry off a piece of furniture which they could not lift in their cooler moments. A striking instance of the tremendous energy of this superadded force occurred in one of the old Scottish wars. A soldier struck a horseman with a battle-axe with such violence, that the weapon at one blow clove down through the rider and his horse, killing both, and then broke a paving-stone beneath.

The common experiment of a few persons lifting another on the tips of their fore-fingers is another instance. Standing around, they all take breath together, and at a given signal they blow under the person to be raised, when he rises like a cork. So striking is the result, and so little is the consciousness of exertion, that the operators often imagine that the person is raised by the breath they blow under him, and not by their fingers. It is obvious, however, that the sole use of the breath is to be a signal, and by the formality of the preparation, to concentrate their thoughts intently on the desired action.

Here, then, we have the power for producing the spiritual manifestations, viz.: muscular power without volition, and without distinct consciousness. It now remains to show how involuntary power can produce intelligent actions, which is quickly done.

The most striking law of this involuntary force is its tendency to execute whatever motions the mind dwells upon, even contrary to the will. Who has not felt the irresistible disposition to move his head when sitting for a daguerreotype, simply from fixing it so strongly in mind that that motion must not be made. So in the above cases of excitement, the superadded force comes in to execute the movements upon which the mind is intent: hence it coincides with volition. The case is the same in a thousand instances in life where a vivid conception of an action causes an unconscious imitation of it. It is seen also in skilled musicians, in whom the mere desire to have a certain note prompts the requisite motion oi the fingers without any consciousness of volition, and it is remarkable that this involuntary style of action gives a more delicate and perfect execution than acts of mere will.

Now the Spiritualists have the merit of having demonstrated that this involuntary power may be separated from the voluntary, and made to act alone; and also that the thought or wish of any motion is as efficient as willing the motion. This is the whole mytery of involuntary writing and tipping. Any sensitive person may try the experiment for himself. Take a pencil in the hand, and without any support for the arm, hold the point lightly on a sheet of paper until the hand begins to twitch and tremble with nervousness and fatigue—a little superstitious awe will help—then looking earnestly at the pencil, picture in your fancy vividly the letters you wish to produce. If you are of a nervous temperament, you will now feel an involuntary impulse of the hand in the requisite directions, and by perseverance and repetition, you may in a little time become a writing medium, a telegraph operator for the devil, as Beecher would say, but really, one over whose muscles fancy has usurped the place of will.

We have proved this by actual experiment, and have been able ourselves to write involuntary communications. Table tipping is still easier.

Since writing the above we see by the journals that Dr. Carpenter, of England, has put forth an essay in which he proves that other acts of the mind than the will may control the muscles. We have also just received a letter from Dr. John C. Norton, a highly intelligent of physician of Illinois, in which he says: "In regard to the writing I have probed the matter to the very bottom. I have been a writing medium, and can demonstrate by an analysis of my own mind while engaged in receiving communications, that the spirits of the dead are not at all concerned in it. I do not take the ground that it is all imposture; in fact I know better. *The will has nothing to do with the actions performed, and yet they are all the work of the mind.*"

We are perfectly aware that unexplainable stories are every day told, but they vary of two things; first, of phenomena not rigidly tested, and secondly, of second-hand statements. We have in our investigations detected eye-witnesses of the very highest integrity, in egregious false statements in consequence of their excitement.

In conclusion, we give it as our own impression, that the claim of "spirituality" for the "manifestations," is an unmitigated humbug, and we are willing to test it with any decent medium that dare try it. We will ask twenty plain and fair questions, and we defy any medium in or out of Michigan to answer them all correctly, either by writing, rapping, or tipping; and we will set a suitable table in our room, and after we have taken the proper measures to prevent the application of muscular action and mechanical force, we defy all the spirits out of Pandemonium to move it a single foot.

